# Genome‐wide transcriptome signatures of ant‐farmed *Squamellaria* epiphytes reveal key functions in a unique symbiosis

**DOI:** 10.1002/ece3.8258

**Published:** 2021-10-26

**Authors:** Yuanshu Pu, Alivereti Naikatini, Oscar Alejandro Pérez‐Escobar, Martina Silber, Susanne S. Renner, Guillaume Chomicki

**Affiliations:** ^1^ German Centre for Integrative Biodiversity Research (iDiv) Halle‐Jena‐Leipzig Leipzig Germany; ^2^ South Pacific Regional Herbarium Institute of Applied Sciences The University of the South Pacific Suva Fiji; ^3^ Royal Botanic Gardens Kew Richmond UK; ^4^ Systematic Botany and Mycology Department of Biology University of Munich (LMU) Munich Germany; ^5^ Department of Biology Washington University Saint Louis Missouri USA; ^6^ Ecology and Evolutionary Biology School of Biosciences University of Sheffield Sheffield UK

**Keywords:** ant agriculture, de novo transcriptomics, mutualism, *Squamellaria*, symbiosis

## Abstract

Farming of fungi by ants, termites, or beetles has led to ecologically successful societies fueled by industrial‐scale food production. Another type of obligate insect agriculture in Fiji involves the symbiosis between the ant *Philidris nagasau* and epiphytes in the genus *Squamellaria* (Rubiaceae) that the ants fertilize, defend, harvest, and depend on for nesting. All farmed *Squamellaria* form tubers (domatia) with preformed entrance holes and complex cavity networks occupied by *P*. *nagasau*. The inner surface of the domatia consists of smooth‐surfaced walls where the ants nest and rear their brood, and warty‐surfaced walls where they fertilize their crop by defecation. Here, we use RNA sequencing to identify gene expression patterns associated with the smooth versus warty wall types. Since wall differentiation occurred in the most recent common ancestor of all farmed species of *Squamellaria*, our study also identifies genetic pathways co‐opted following the emergence of agriculture. Warty‐surfaced walls show many upregulated genes linked to auxin transport, root development, and nitrogen transport consistent with their root‐like function; their defense‐related genes are also upregulated, probably to protect these permeable areas from pathogen entry. In smooth‐surfaced walls, genes functioning in suberin and wax biosynthesis are upregulated, contributing to the formation of an impermeable ant‐nesting area in the domatium. This study throws light on a number of functional characteristics of plant farming by ants and illustrates the power of genomic studies of symbiosis.

## Graphical abstract

We performed the first transcriptomic analysis of the newly discovered obligate farming symbiosis involving ants farming plants. Our analyses reveal a number of candidate genes associated with the differentiation of the plant tissue for ant nesting and fertilization.

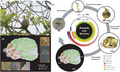

## 1 INTRODUCTION

Diverse cultivation mutualisms have evolved across the tree of life, for instance in systems involving snails (Silliman & Newell, 2003), crabs (Thurber et al., 2011), amoeba (Brock et al., 2011), fungi (Pion et al., 2013), three‐toed sloths (Pauli et al., 2014), and damselfish (Hata & Kato, 2006). In contrast to these cultivation mutualisms, true agriculture is defined by four components, namely habitual planting, cultivation, harvest, and dependence of the farmer on the crop (Mueller et al., 2005). Outside of humans, agriculture is restricted to social insects (ants, termites, beetles) that cultivate fungi (Aanen et al., 2002; Farrell et al., 2001; Mueller et al., 2005; Schultz & Brady, 2008) and ants that cultivate plants (Chomicki, 2022; Chomicki & Renner, 2016a). The iconic symbioses of ants farming fungi have been studied for decades, with foci on their evolution (Aanen et al., 2002; Farrell et al., 2001; Mueller & Gerardo, 2002; Mueller et al., 1998; Schultz & Brady, 2008), stability (Aanen et al., 2009; Poulsen & Boomsma, 2005), cultivation behavior (Biedermann et al., 2009; Fernández‐Marín et al., 2009; Katariya et al., 2017; Van Bael et al., 2009), genomics (Nygaard et al., 2016; Poulsen et al., 2014; Vanderpool et al., 2018), and control of fungal parasites (Currie et al., 2003; Li et al., 2018). Ant gardens, generalist cultivation mutualisms in which arboreal ants plant the seeds of various epiphytic species to strengthen their carton nest, have been known for 120 years (Ule, 1901), but do not involve true agriculture because they are neither reciprocally obligate nor always involve crop‐derived food.

Genomic approaches to insect agriculture have revealed reciprocal biochemical and genetic adaptations. For example, in leaf‐cutting ants, genes in the ants’ arginine biosynthetic pathway have been lost because arginine is supplied by the ants’ fungal crop. Conversely, the fungal crops show changes in chitin synthesis and loss of enzymes to degrade lignin (Nygaard et al., 2011, 2016). And in *Termitomyces* fungi cultivated by *Macrotermes* termites, the degradation of complex plant carbohydrates occurs via the cooperation of both the fungus and the termites, as well as their bacterial gut microbiomes (Poulsen et al., 2014). In ant/plant symbioses, genome sequencing of *Pseudomyrmex* ant species that independently evolved obligate nesting in mutualistic plants revealed positive selection of many genes involved in nervous system functioning, associated with their aggressive behavior toward herbivorous insects (Rubin & Moreau, 2016). However, to our knowledge, no genomic study has focused on the plant partners involved in these symbioses, likely because of the difficulty of working with large plant genomes without reference.

In the present study, we apply transcriptomics to the plant partner in an obligate insect agriculture system discovered in Fiji. This system involves the symbiosis between the ant *Philidris nagasau* (Dolichoderinae) and epiphytes in the genus *Squamellaria* (Rubiaceae) that the ants fertilize, defend, harvest, and depend on for nesting (Chomicki et al., 2019, 2020; Chomicki & Renner, 2016a, 2016b). Fiji harbors nine species of *Squamellaria* of which six are obligately ant farmed, while three form facultative mutualisms with a wide range of ants, with the obligate ant‐farmed species forming a clade, the others a grade (Chomicki & Renner, 2016a, 2016b; Chomicki et al., 2019). All *Squamellaria* species, like other species in the Hydnophytinae clade of the Rubiaceae, form a large tuber with self‐formed entrance holes and complex cavity networks in which ants nest (Chomicki & Renner, 2017, 2019). Ant‐farmed *Squamellaria* also offer food rewards to their ant partners, and in return, the ants fertilize their hosts (their “crop”) by defecation, disperse their seeds, protect the seeds from predators, and defend the plant against herbivores (Chomicki et al., 2016, 2020; Chomicki & Renner, 2016a, 2019). The single ant species involved in the *Squamellaria* farming symbiosis, *P*. *nagasau*, besides displaying numerous of specialized behaviors, also evolved the ability to evaluate and optimize the yield of its crop (in terms of food rewards) by cultivating its crop in high‐light conditions (Chomicki et al., 2020).

**FIGURE 1:**
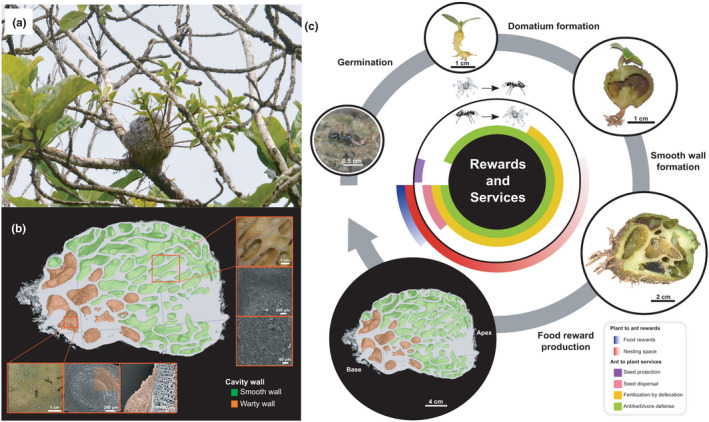
Domatium tissue differentiation in ant‐farmed *Squamellaria*. (a) *Squamellaria imberbis*, Vanua Levu island, Fiji. (b) Longitudinal section from computed‐tomography scanning and scanning electron micrographs showing the differentiated smooth and warty walls inside the farmed *Squamellaria* domatium. (c) Rewards and services exchanged between *Squamellaria* and *Philidris nagasau* ant through the plant ontogeny, highlighting the development of warty (orange) and smooth walls (green) inside the domatium. The tuber tissue is shown in gray. (b) and (c) are taken from Chomicki and Renner (2019), with permission from Wiley‐Blackwell (UK)

An important trait of all farmed *Squamellaria* species is the differentiation of the inner domatium walls (Chomicki & Renner, 2019). Nonfarmed *Squamellaria* exhibit an intermixing of warty‐surfaced and smooth‐surfaced sections in their domatia, with low nitrogen uptake efficiency. In contrast, the inner cavity surface of farmed *Squamellaria* is differentiated into smooth areas that are impermeable to water or solutes and hyperabsorptive warty areas that are specialized for nitrogen uptake (Chomicki & Renner, 2019; Figure 1). This led us to hypothesize that genes involved in suberin or wax production might be upregulated in the smooth tissues, while genes involved in nitrogen uptake might be upregulated in the warty tissues on which the ants defecate. To test these hypotheses, we here use genome‐wide transcriptomes of the inner domatium walls of an obligately farmed *Squamellaria* species (*S*. *imberbis*, Figure 1a). Using de novo RNA sequencing (RNA‐seq), we aimed to answer the following questions: (i) What classes of genes are upregulated in warty‐surfaced versus smooth‐surfaced domatium walls? (ii) Are genes encoding for biosynthesis or regulation of wax or suberin compounds upregulated in smooth walls? And because warts are functionally analogous to roots (iii) are root development genes upregulated in warty walls?

## 2 METHODS

### 2.1 Study site and samples preparation

Samples of three *S*. *imberbis* individuals were collected on Vanua Levu island in Fiji, along the Cross Island road before the bifurcation to Nabouwalu and Labasa in July 2018. Samples were immediately placed in RNAlater (Sigma‐Aldrich) until their extraction <72 h later. Plants were identified by the senior author (G.C.), following the taxonomy of Chomicki and Renner (2016b) and documented by the herbarium voucher G. Chomicki, J. Aroles, A. Naikatini 50 (M). Three tissue samples per plant were collected: (1) the warty surface of the basal wall of the domatium (1 mm thickness); (2) the smooth surface in the apex cavities of the domatium (1 mm thickness); and (3) the tuber tissue under the wall surface. Tuber tissue (i.e., domatium tissue without any differentiation, shown in gray in Figure 1b) was used as a control. All individuals sampled were healthy, mature plants of a similar size (domatium diameter *c*. 15 cm), each inhabited by *P*. *nagasau* ants (coming from distinct ant colonies). RNA extraction was performed using a NucleoSpin RNA Plant Mini Kit (Macherey‐Nagel, Düren, Germany) following the manufacturer's protocol.

### 2.2 Sequencing and dataset preparation

For each RNA sample, a strand‐specific cDNA library (library type: RF, the first read of a fragment pair is from the opposite strand) with mRNA selection using poly‐A tail enrichment was constructed and sequenced using the Illumina HiSeq 2500 Genome Sequencer (Illumina, San Diego). Libraries were sequenced to a depth of at least 37 million pairs of reads of 150‐bp length. Library preparation and sequencing was performed by the company Eurofins Genomics (Konstanz, Germany).

Raw reads were preprocessed by performing adapter and base trimming, using a quality Phred score of 30 as implemented in the software Trim Galore! v0.6.4 (Krueger, 2015). FastQC v0.11.9 (Andrews, 2010) was used to assess the quality of trimmed reads, with the per‐base sequence quality scores of all sequences higher than 34. The raw sequence datasets are available in the Short Read Archive of the NCBI repository (https://www.ncbi.nlm.nih.gov/sra) under the BioProject accession number PRJNA734727.

### 2.3 Transcriptomic analyses

#### 2.3.1 De novo assembly and quantitation

*De novo* assembly of all the trimmed RNA‐seq data was conducted using Trinity v2.8.5 (Grabherr et al., 2011). All sequences of the three tissue types were combined to obtain a single complete assembly. Then the abundance of each sample transcript was independently estimated on both isoform and gene level, using the alignment‐free method implemented in the software Salmon v0.14.1 (Patro et al., 2017). When estimating gene counts, trimmed mean of M values (TMM) were used for normalization, and the scaled Transcripts‐per‐million (TPM) method in tximport (Soneson et al., 2015) was used to exclude effects of differential transcript usage caused by differences in isoform lengths. All estimates of the nine samples (three tissue types from three plant individuals) were used to construct matrices of raw counts, TPM expression values (not cross‐sample normalized), and TMM‐normalized expression values.

#### 2.3.2 Quality check

Before further analyses, relationships among different types of samples and biological replicates were examined by Spearman's rank correlation coefficient and principal component analysis (PCA) using the TMM‐normalized expression values. Although Spearman's rank correlation coefficient did not show that the replicates are more correlated within samples, PCA indicated that each type of tissue clustered together using the first two principal components (Figure S1).

#### 2.3.3 Differential expression analyses

Among the three types of samples (warty surfaces, smooth surfaces, tuber tissue), differential pairwise expression analyses were conducted using the software edgeR v3.28.0 (McCarthy et al., 2012; Robinson et al., 2010) on the raw count matrices generated in the quantitation step, on both gene and isoform levels. For each pair of comparison, log fold change (log FC) and the False Discovery Rate (FDR) were calculated. On the gene level, all genes that had *p*‐values <.001 and were at least 2^2^‐fold differentially expressed (DE) in a comparison between any two types of samples were extracted to be further investigated. The TMM‐normalized expression values of these DE genes were log2‐transformed and median centered to plot a clustered heatmap of DE genes versus sample replicates. All DE genes are reported in Table S1.

#### 2.3.4 Functional annotation

Coding region prediction for all DE Trinity gene assemblies was conducted using TransDecoder v5.5.0 (Haas et al., 2013) with a threshold of 100 amino acids as the minimum length of ORF. Then OrthoFinder v2.3.3 (Emms & Kelly, 2015, 2019) was used to search for orthologs of the predicted DE peptides in other model species, including proteomes of *Arabidopsis thaliana* (Brassicaceae), *Coffea canephora* (Rubiaceae), and *Solanum lycopersicum* (Solanaceae). Finally, gene ontology annotation data of *A*. *thaliana* were acquired from PANTHER v14.0 (Thomas et al., 2003) and further annotated to the orthologous genes of *S*. *imberbis*. The annotation of all DE transcripts is reported in Table S2.

#### 2.3.5 Gene set enrichment analysis

To build a customized annotation database of *S*. *imberbis*, we annotated the complete transcriptomic assembly as described in section 2.3.4 and then used Gene Ontology terms in Biological Processes (GOBP) of the *Arabidopsis* orthologs to annotate the corresponding *S*. *imberbis* genes. Next, GeneSCF v1.1 (Subhash & Kanduri, 2016) was used for GOBP enrichment analysis of each group of differentially expressed genes in each of the three tissue types. All significantly enriched GO terms are marked in Table S2, and detailed enrichment statistics are given in Table S3.

## 3 RESULTS

### 3.1 Most differentially expressed genes are upregulated in warty inner domatium tissues

**FIGURE 2:**
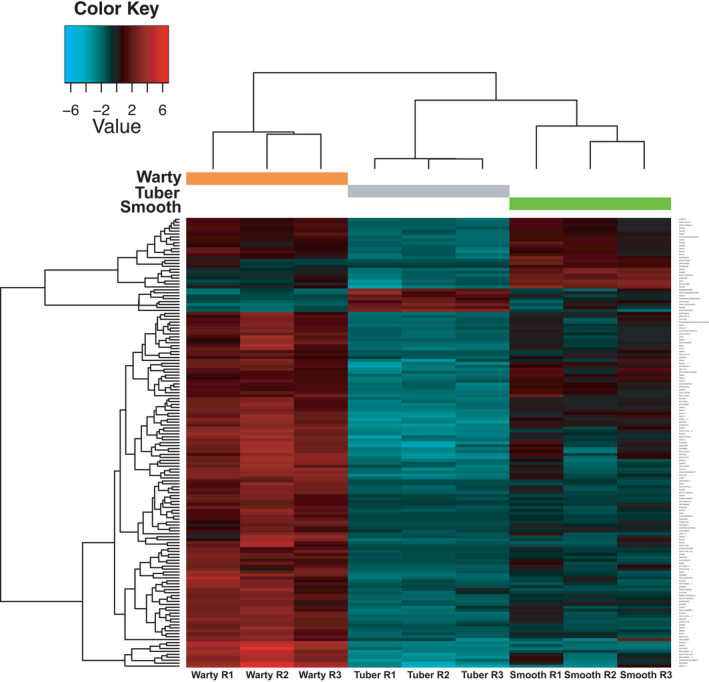
Heatmap of the expression level of 153 Trinity genes that are expressed differentially in at least one kind of tissue, predicted to be coding sequences and annotated successfully to genes of *Arabidopsis thaliana*. Gene names are composed of a prefix of “Si” representing *Squamellaria imberbis* and the respective *A*. *thaliana* gene name annotated to the Trinity gene

Sequencing statistics are summarized in Table 1. Among the 737,226 assembled transcripts, 962 genes were found differentially expressed in at least one comparison between two tissue types (false discovery rate [FDR] <0.001, see Table S1 for statistics of DE analysis of all annotated genes in this study). Comparing warty‐surface tissue and tuber tissue (the control) revealed that 859 genes were significantly more expressed in warts and 44 genes were significantly more expressed in tubers. Comparing warty tissue and smooth tissue showed that 78 genes were significantly more expressed in warty‐surface tissue and 9 were significantly more expressed in smooth‐surface tissue. Comparison of smooth tissue and tuber tissue indicated that 97 genes were significantly more expressed in the former and 5 more in the latter. Among all 3 tissue types, 66 genes were most expressed in warty tissue, 3 in smooth tissue, and 4 in tuber tissue. In addition, 57 genes were least expressed in tuber tissue. No genes were least expressed in warty or smooth tissue compared to the control. Of the 962 differentially expressed genes, 153 could be annotated successfully to genes of *A*. *thaliana* (Figure 2).

**TABLE 1:** Summary of RNA‐sequencing quality

Sample	Raw reads	Clean reads	Clean bases (bp)	Q20 (%)	Q30 (%)	GC (%)
Warty‐R1	88,949,600	88,276,350	12,390,576,293	99.99	98.42	44.5
Warty‐R2	79,415,698	78,997,816	11,111,828,166	99.99	98.76	44.5
Warty‐R3	85,099,860	84,474,992	11,835,679,753	99.99	98.49	47.0
Smooth‐R1	94,434,986	93,791,780	13,156,305,731	99.99	98.50	43.5
Smooth‐R2	86,455,782	85,893,898	12,046,177,689	99.99	98.68	44.0
Smooth‐R3	84,498,192	83,709,338	11,740,992,664	99.99	98.21	45.0
Tuber‐R1	97,776,246	97,054,254	13,613,004,742	99.99	98.47	42.5
Tuber‐R2	75,942,466	75,465,906	10,501,410,231	99.99	98.60	42.0
Tuber‐R3	86,450,798	85,906,544	12,059,318,147	99.99	98.65	42.5

Note

“Warty” refers to warty‐walled inner domatium tissue; “smooth” refers to smooth‐walled inner domatium tissue; “tuber” refers to nondifferentiated domatium tissue, which served as a control. Each sample comes from a different plant individual.

### 3.2 Genes functioning in wax and suberin biosynthesis are upregulated in smooth inner domatium tissues

**FIGURE 3:**
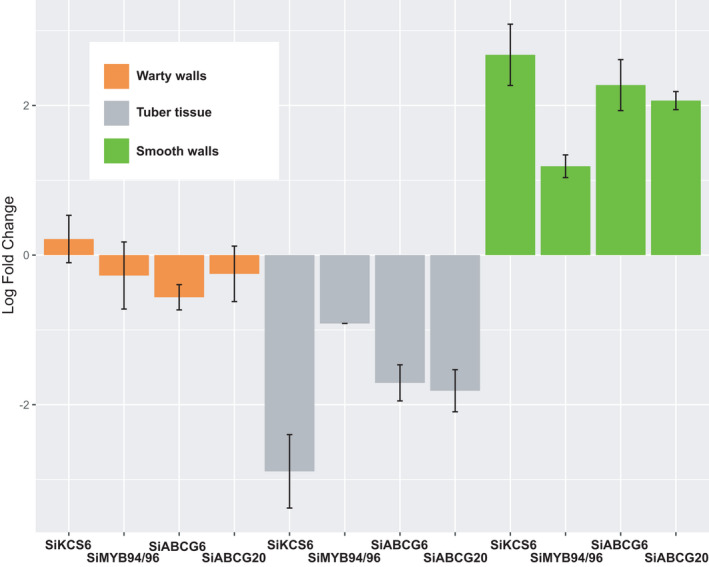
Expression level of wax (*KCS6* and *MYB94*/*96)* and suberin (*ABCG* gene family) biosynthesis genes in domatium tissue, showing upregulation in smooth cavities where they are essential in the formation of the impermeable coating of smooth walls

Of the 97 orthologues significantly more expressed in smooth tissues, 2 were annotated to *Arabidopsis KCS6* and the *MYB94* and *MYB96* genes, which function in wax biosynthesis (Figure 3; Lee et al., 2014; Millar et al., 1999; Seo et al., 2011) and 2 to the ATP‐Binding Cassette genes *ABCG6* and *ABCG20*, which function in suberin biosynthesis (Figure 3; Yadav et al., 2014). The Gene Ontology term suberin biosynthetic process (GO: 0010345) was significantly enriched with a *p*‐value of 4.78E‐05 and FDR of 6.21E‐04. Besides, a series of GO biological processes related to wax/suberin biosynthesis (GO:0006869~lipid transport, GO:0019915~lipid storage, GO:0042761 ~ very long‐chain fatty acid biosynthetic process, and GO:0010208 ~ pollen wall assembly) were also significantly enriched in smooth surfaces (*p* < .02, FDR < 0.03).

### 3.3 Genes functioning in auxin metabolism and transport, and auxin response genes are upregulated in warty inner domatium tissues

**FIGURE 4:**
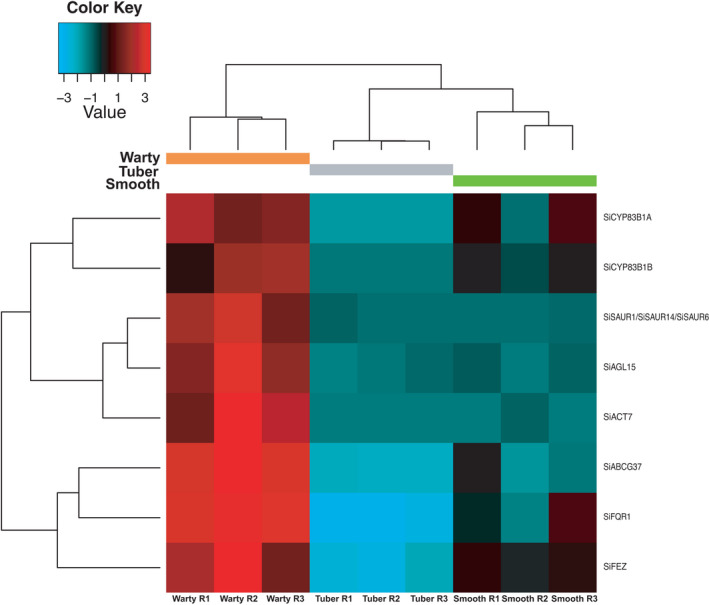
Heatmap of the expression level of Trinity genes of *Squamellaria imberbis* annotated to *Arabidopsis thaliana* genes involved in auxin metabolism and transport, or responses to auxin signals. *Si* stands for *Squamellaria imberbis* and gene name abbreviations are as follows: *CYP83B1*: *Cytochrome P450 Family 83 Subfamily B Polypeptide 1*, *SAUR1*/*6*/*14*: *Small Auxin Upregulated RNA 1*/*6*/*14*; *AGL15*: *Agamous*‐*Like 15*; *ACT7*: *Actin gene family 7*; *ABCG37*: *ATP*‐*Binding Cassette* 37; *FQR1*: *Flavodoxin*‐*Like Quinone Reductase 1*; *FEZ*: a *NAC* Domain Transcription Factor

Eight Trinity gene orthologues significantly more expressed in warts were involved in processes related to auxin metabolism and transport or the response to auxin signals (Figure 4, Table S1). Among these orthologues, Cytochrome P450 Family 83 Subfamily B Polypeptide 1 (*CYP83B1*; we identified two copies, noted *SiCYP83B1 A* and *B*) is involved in the auxin biosynthetic process (Bak et al., 2001), *ABCG37* is involved in auxin transport and cellular response to auxin (Růžička et al., 2010), and Flavodoxin‐Like Quinone Reductase 1 (*FQR1*), the *NAC* Domain Transcription Factors *FEZ*, Actin gene family 7 (*ACT7*), Agamous‐Like 15 (*AGL15*), and Small Auxin Upregulated RNA 1/6/14 (*SAUR1*/*SAUR6*/*SAUR14*) are involved in responses to auxin (Jain et al., 2006; Laskowski et al., 2002; Willemsen et al., 2008).

### 3.4 Genes functioning in root development are upregulated in warty domatium tissues

**FIGURE 5:**
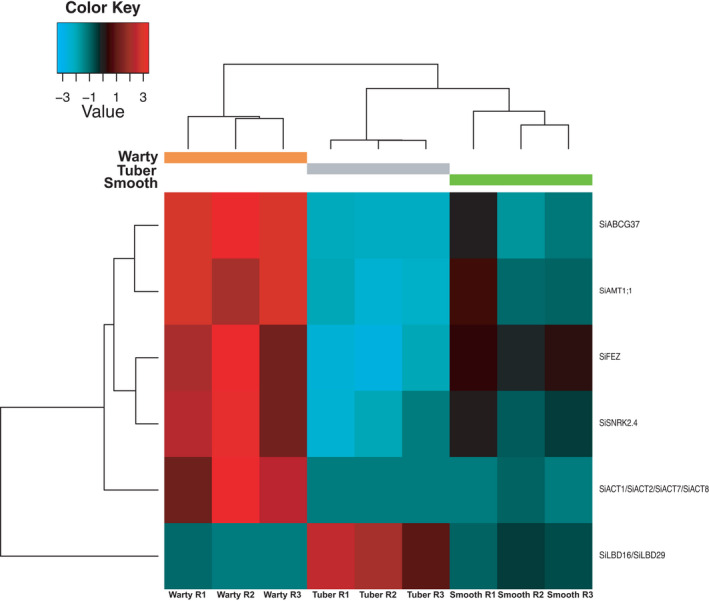
Heatmap of the expression level of Trinity genes of *Squamellaria imberbis* annotated to *Arabidopsis thaliana* genes involved in root development. *Si* stands for *Squamellaria imberbis* and gene name abbreviation are as follows: *ABCG37*: *ATP*‐*Binding Cassette* 37; *AMT1*;*1*: *Ammonium Transporter 1*;*1*; *FEZ*: a *NAC* Domain Transcription Factor; *NRK2*.*4*: *Sucrose Nonfermenting 1*‐*Related Protein Kinase 2*.*4*; ACT1/ACT2/ACT7/ACT8: Actin 1, 2, 7, 8; LBD16/LBD29: Lateral Organ Boundaries‐Domain 16 and 29

Some of the auxin‐related genes upregulated in warty tissues are also involved in root development, that is, *ABCG37*, *ACT7*, and the *NAC* Domain Transcription Factors *FEZ* (Růžička et al., 2010; Willemsen et al., 2008). Other genes related to root development that were significantly more expressed in warts included Ammonium Transporter 1;1 (*AMT1*;*1*), Sucrose Nonfermenting 1‐Related Protein Kinase 2.4 (*SnRK2*.*4*) and more members of the Actin gene family (*ACT1*, *ACT2*, and *ACT8*) (Lima et al., 2010; McLoughlin et al., 2012). In total, five Trinity genes related to root development were significantly more expressed in warty tissue. Genes significantly more expressed in regular tuber tissue were annotated to the Lateral Organ Boundaries‐Domain 16 and 29 (*LBD16* and *LBD29*) (Figure 5). However, no GOBP related to root development was significantly enriched among DE genes.

### 3.5 Genes functioning in biotic and abiotic stress responses are upregulated in warty inner domatium tissues

**FIGURE 6:**
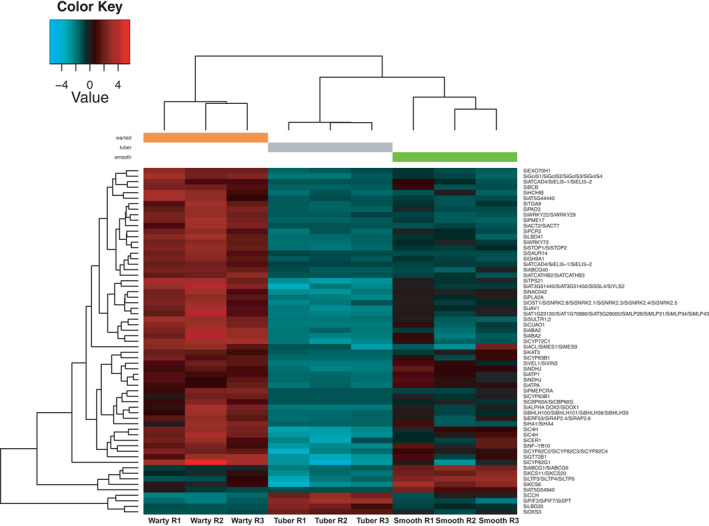
Heatmap of the expression level of Trinity genes of *Squamellaria imberbis* annotated to *Arabidopsis thaliana* genes involved in response to biotic and abiotic stress, including defense against fungal and bacterial pathogens

A total of 54 Trinity genes that are orthologous to *Arabidopsis* genes involved in defense and response to external stimuli were significantly more expressed in warty surfaces (Figure 6). Examples are genes that function in response to microbes including fungal pathogens (*SSL4*; Sohani et al., 2009), Gram‐negative bacteria (Pectin Methylesterase 17 (*PME17*); Bethke et al., 2014), herbivores (Terpene Synthase 21 (*TPS21*); Hong et al., 2012), cold or heat stress (Galactinol Synthase 1/2/3 (*GOLS1*/*2*/*3*), Abscisic Acid 2 (*ABA2*); Taji et al., 2002; Baron et al., 2012), and osmotic stress (Snf1‐related protein kinases *SnRK2*.*4*; McLoughlin et al., 2012). One large group of these genes are involved in the Jasmonic acid metabolic process, the Jasmonic acid‐mediated signaling pathway, and biological responses to Jasmonic acid stimuli (Pré et al., 2008; Sohani et al., 2009; Zhu et al., 2010) and are annotated to Basic Chitinase (*HCHIB*), Strictosidine Synthase‐Like 4 (*SSL4*), Yellow‐Leaf‐Specific Gene 2 (*YLS2*), Ethylene Responsive Factors 53 and 54 (*ERF53*), a transcription factor family (*RAP2*), Jasmonate‐Associated VQ Motif Gene 1 (*JAV1*), *ABCG40*, Methyl Esterase 1 and 9 (*MES1* and *MES9*), and Acetone‐Cyanohydrin Lyase (*ACL*). Nine GO biological processes related to defense and stress response are significantly enriched among DE genes (all *p*‐value < .05, FDR < 0.1), including defense response to virus, bacterium, fungus, nematode, and other organisms (GO: 0009615, 0009617, 0050832, 0009624, 0098542), biosynthesis of terpene and camalexin (GO: 0046246, 0010120), jasmonic acid and ethylene‐dependent systemic resistance (GO: 0009871), and cellular response to salicylic acid stimulus (GO: 0071446).

### 3.6 Genes functioning in nitrogen uptake and transport are upregulated in warty inner domatium tissues

**FIGURE 7:**
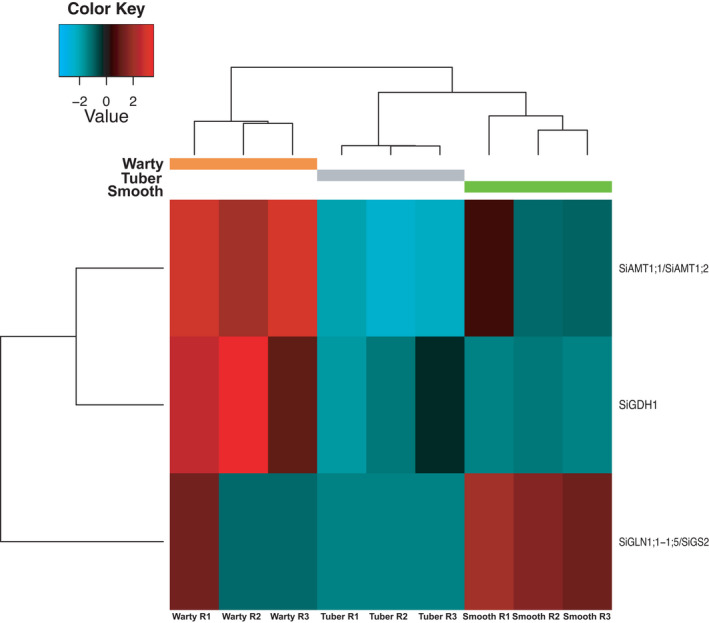
Heatmap of the expression level of Trinity genes of *Squamellaria imberbis* annotated to *Arabidopsis thaliana* genes involved in mineral and organic nitrogen uptake and transport. *Si* stands for *Squamellaria imberbis* and gene name abbreviations are as follows: *AMT1*;*1*/*AMT1*;*2*: *Ammonium Transporter 1*;*1* and *1*;*2*; *GDH1*: *Glutamate dehydrogenase 1*; *GLN1*;*1*−*1*;*5*/*GS2*: *Glutamine synthase 1*;*1*

Two Trinity genes that are orthologues of *A*. *thaliana* genes related to nitrogen metabolism and transport were significantly more expressed in warty tissues (Figure 7), namely *Ammonium Transporter 1*;*1* (*AMT1*;*1*), *AMT1*;*2* and *Glutamate dehydrogenase 1* (*GDH1*), which function in ammonia assimilation and ammonium transport (Mayer & Ludewig, 2006; Meng et al., 2016). We also identified the significant enrichment of three GOBP related to ammonium transport (GO: 0015843~methylammonium transport, GO: 0015696~ammonium transport, and GO: 0072488~ammonium transmembrane transport).

## 4 DISCUSSION

### 4.1 Upregulation of wax and suberin genes is associated with the formation of clean and sturdy nesting sites inside domatia

**FIGURE 8:**
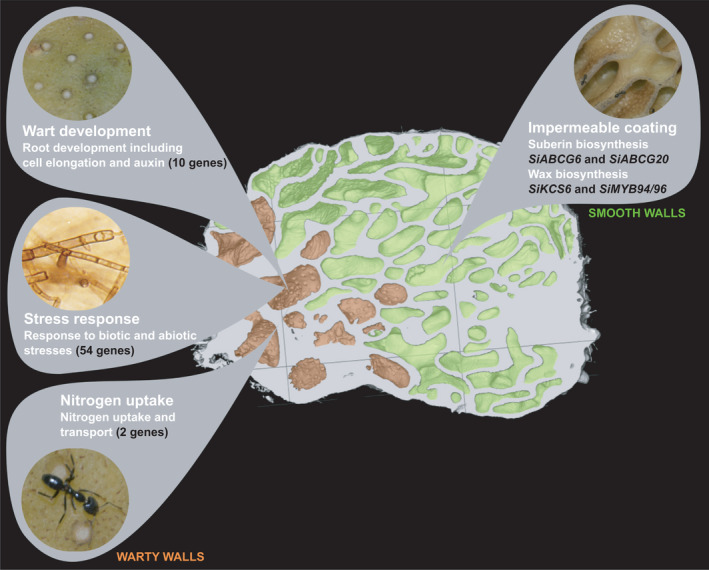
Model showing the main functions underlying the genes expressed in warty versus smooth walls in *Squamellaria imberbis* domatia. In warty walls, our transcriptome analysis revealed that putative genes are involved in wart development and possibly spacing (root transcriptional regulators, cell elongation factors and genes involved in auxin transport and response); genes are involved in the response to biotic and abiotic stresses (including defense against fungal and bacterial pathogens); and finally, genes that likely play a role in the uptake of ant‐derived nitrogen. In smooth walls, upregulation of wax and suberin genes likely acts in concert to generate the impermeable coating on these walls for ant nesting

This transcriptome analysis revealed that genes upregulated in the inner walls of ant‐housing plant tubers (domatia) function in the biosynthesis of wax and suberin (Figures 3 and 8). Experiments in the field have shown that these surfaces do not absorb water or solutes, which suggested an impermeable coating (Chomicki & Renner, 2019). Both suberin and waxes are hydrophobic impermeable materials. For suberin, a group of *ABCG* half‐transporters (*ABCG1*,*6*,*16*,*20*) has been shown to control water and solute movement, typically in roots and seed coats (Yadav et al., 2014). For wax, the *KCS6* gene (also known as *CUT1*) is involved in the production of cuticular wax, which makes up all stem wax components in *Arabidopsis* (Millar et al., 1999). The upregulation of these genes in a certain section of the domatium cavities allows the formation of a sturdy nesting site for *Philidris nagasau* ants.

Besides forming hydrophobic barriers, wax also functions as a defense against bacterial and fungal pathogens (Jenks et al., 1994; Wang et al., 2020; Zhang et al., 2019) and insect herbivores (Eigenbrode & Espelie, 1995), and suberin is also thought to play an important role in limiting pathogen access (Pollard et al., 2008; Thomas et al., 2007). Thus, the coating of smooth walls with wax and suberin likely reduces bacterial and fungal pathogen growth (Zhang et al., 2019). Another potential role of the wax/suberin coating is the protection of wall tissue against damage from the claws of the ants’ tarsae; the constant high concentration of workers in these cavities might otherwise incur a high mechanical cost to the domatium tissue. Thick cuticles inside a domatium are also seen in unrelated species, for instance in the leaf domatia of *Hirtella physophora* (Leroy et al., 2008). Further work is needed to test these potential roles in defense against pathogens and mechanical stress.

### 4.2 Warts are functionally analogous to adventitious roots

Auxin plays many roles related to plant growth and body formation, from root development to shoot apical dominance and branching regulation, flowering, wound response, and fruit growth (Woodward & Bartel, 2005). It also controls adventitious root development in response to environmental signals (Gonin et al., 2019). In this study, many of the genes upregulated in warty inner domatium tissues relate to auxin transport and have orthologues expressed in *A*. *thaliana* roots (Figures 4, 5 and 8). For example, *ABCG37* is expressed in the lateral root cap and epidermis (Růžička et al., 2010); and the *NAC* Domain Transcription Factors *FEZ* is expressed in root cap stem cells, promoting periclinal root cap‐forming cell division (Willemsen et al., 2008). That warts inside domatium cavities may be analogous to roots was originally hypothesized by Beccari (1884–86) at the end of the 19th century, while he investigated the closely related genus *Myrmecodia*, and field experiments on nutrient uptake in two *Squamellaria* species have demonstrated the warts’ hyperabsorptive function (Chomicki & Renner, 2019). The transcriptomic data obtained here now support this function. Warts, thus, appear to be functionally analogous to adventitious roots.

Besides auxin‐related processes that are active during root development (Overvoorde et al., 2010), other genes involved in root development were also upregulated in warty surfaces compared to smooth surfaces and undifferentiated tuber tissues. Among them, multiple isovariants of the Actin gene family regulate different aspects of root development. Mutants of *ACT2* and *ACT8* genes exhibit root hair defects, while a mutant of the *ACT7* gene exhibits root elongation defects (Kandasamy et al., 2009). *Squamellaria* inner domatium warts likewise show cell elongations at their surface (G. Chomicki personal observation), which is consistent with an involvement of *ACT* in wart growth and development. Other genes upregulated in *Squamellaria* warty cavities have orthologues in *Arabidopsis* that affect the root system under particular environmental conditions, such as the Snf1‐related protein kinases *SnRK2*.*4*, which are activated under salt stress (McLoughlin et al., 2012) and *AMT1*;*1*, which increases lateral root initiation and higher order lateral root branching when stimulated by local ammonium supply (Lima et al., 2010).

### 4.3 Defense‐ and stress‐related genes are upregulated to protect permeable warty inner domatium surfaces

Fifty‐four genes upregulated in the warty inner domatium surfaces function in defense and in response to a variety of external stimuli (Figure 6). For example, the *SSL* gene family plays key roles in bacteria‐, fungus‐ and wounding‐induced plant defense mechanisms (Sohani et al., 2009); pectin methylesterases (*PMEs*) function mainly in immunity against gram‐negative bacteria such as *Pseudomonas syringae* (Bethke et al., 2014); and the induction of Terpene Synthase 12 (*TPS21*) results in increased emission of sesquiterpenes, which is among the herbivore‐induced plant volatiles that attract natural enemies of attacking insects and that warn neighboring plants (Hong et al., 2012; War et al., 2011). This highlights an important aspect of the *Squamellaria*/*Philidris nagasau* farming symbiosis: While the smooth surfaces are covered by a tough hydrophobic suberin and wax coating that reduces pathogen entry and growth, the warty surfaces are hyperabsorptive and, thus, are potential entry points for pathogens. Moreover, the ants defecate on the warty surfaces (Chomicki & Renner, 2016a), likely making these nutrient‐rich areas a substrate for pathogen growth. That numerous pathogen defense‐related genes are highly expressed in warty surfaces suggests that the plant upregulates chemical defenses to counteract the surface permeability required for nutrient and water absorption.

Two genes upregulated in warts that appear to be particularly important are Galactinol synthase (*GolS*) 1 and 2, which function in response to abiotic stresses such as osmotic and oxidative stress, heat, chilling, and water deprivation. *GolS1* is a heat shock target gene, which is responsible for heat stress‐dependent synthesis of raffinose (Panikulangara et al., 2004), and *GolS2* leads to increased drought resistance (Taji et al., 2002) and oxidative stress (Nishizawa et al., 2008). In *Squamellaria* domatia, the concentrated defecation of ants on the warty surfaces may create a hypertonic environment, and tolerance of oxidative or hypertonic stress may be crucial.

## 5 CONCLUSION

The obligate mutualism between *Squamellaria* and *Philidris* ants has led to the differentiation of the plant's inner domatium walls into warty surfaces for defecation and smooth surfaces for nesting. This transcriptome analysis revealed genes functioning in nutrient uptake and defense against pathogens upregulated in the warty surfaces, contributing to the plant's ability to use nutrients from the ants’ targeted defecation while minimizing pathogen entry (Figure 8). It also identified genes functioning in wax and suberin biosynthesis upregulated in the smooth surfaces, contributing to the formation of clean nesting sites. This work paves the way for comparative genomic studies of symbiosis‐related genes across the Hydnophytinae clade, the most species‐rich clade of ant‐plants worldwide.

## CONFLICT OF INTEREST

The authors declare no competing interest.

## AUTHOR CONTRIBUTIONS

**Yuanshu Pu:** Conceptualization (supporting); Formal analysis (lead); Investigation (equal); Methodology (equal); Writing‐original draft (lead). **Alivereti Naikatini:** Investigation (supporting); Resources (equal). **Oscar Alejandro Pérez‐Escobar:** Formal analysis (supporting); Methodology (equal); Supervision (supporting); Writing‐review & editing (equal). **Martina Silber:** Investigation (equal); Methodology (equal). **Susanne S. Renner:** Funding acquisition (equal); Resources (equal); Supervision (supporting); Writing‐original draft (supporting); Writing‐review & editing (equal). **Guillaume Chomicki:** Conceptualization (lead); Funding acquisition (equal); Project administration (lead); Resources (equal); Supervision (lead); Writing‐original draft (equal).

## Supporting information

Figure S1

Table S1

Table S2

Table S3

## Data Availability

RNA‐seq sequences are available in GenBank (https://www.ncbi.nlm.nih.gov/genbank/) at the accession number SRA (BioProject accession: PRJNA734727). Assembled RNA‐seq data are available in Figshare (https://doi.org/10.6084/m9.figshare.16722982.v1).
