# Supplementary figures and images for: Genome‐wide transcriptome signatures of ant‐farmed Squamellaria epiphytes reveal key functions in a unique symbiosis
Source: Ecol Evol. 2021 Oct 26;11(22):15882–95. doi: 10.1002/ece3.8258 (PMC8601933; doi:10.1002/ece3.8258)

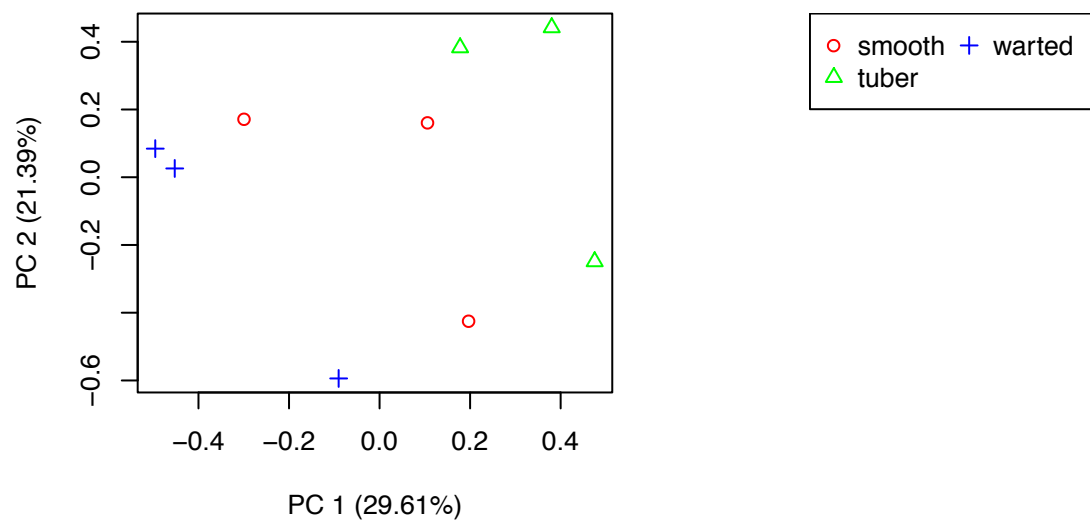

**Figure S1.** Principal component analysis of the RNAseq samples and replicates.

Supplement: Supplementary file 1 — Figure S1 [file ECE3-11-15882-s002.pdf]
